# Abdominal Tuberculosis May Masquerade Many Diseases

**DOI:** 10.4103/1319-3767.77239

**Published:** 2011

**Authors:** Sankappa P. Sinhasan, Rekha B. Puranik, Mohan H. Kulkarni

**Affiliations:** Department of Pathology, Hassan Institute of Medical Sciences, Hassan, Karnataka, India; 1 Department of Pathology, Karnataka Institute of Medical Sciences, Hubli, India

**Keywords:** Adenocarcinoma caecum, chronic nonspecific enteritis, Crohn’s disease, intestinal tuberculosis, intussusceptions, ischemic enteritis

## Abstract

**Background/Aim:**

Intestinal tuberculosis needs to be considered in the differential diagnosis when patients with intestinal pathology are encountered. Tuberculosis can mimic other disease entities like, ischemic enteritis, inflammatory bowel diseases, malignancies, intussusception etc., clinically as well as morphologically in resected intestinal specimens. We aimed to study the various clinical presentations leading to intestinal resection, with identification of different etiological factors by histopathological examination; and to illustrate, discuss and describe the various histopathological features of the lesions in these resected intestinal specimens with clinicopathological correlation.

**Materials and Methods:**

We studied 100 cases of resected intestinal specimens received during September 2002 to December 2003. We totally encountered 22 request forms with clinical suspicion of ileoceocal tuberculosis.

**Results:**

Abdominal tenderness and mass in ileoceocal region were noted in all cases. In many instances, the cases were operated for acute/subacute intestinal obstruction. Clinical and intra-operative diagnoses of tubercular enteritis, in many instances, were finally diagnosed histopathologically as ischemic enteritis (nine cases), chronic nonspecific enteritis (four cases), adenocarcinoma of the caecum, Crohn’s disease, intussusception (each one case), and correctly as intestinal tuberculosis in only six cases.

**Conclusion:**

Tuberculosis can mimic various disease entities, clinically and sometimes morphologically. Vice versa is also true. An increased awareness of intestinal tuberculosis coupled with varied clinical presentations, nonspecific signs and symptoms, difficulties in diagnostic methods and need of early and specific treatment should improve the outcome for patients with this disease.

Tuberculosis was recognized as early as the fourth century B.C. and was known as phthisis, lupus, scrofula or Pott’s disease, until identity was established by Robert Koch in 1882. Hippocrates stated that, “Phthisis persons die if diarrhea sets in and it is a mortal symptom.” The severity of intestinal tuberculosis was known even at that time.[1]

Tuberculosis is a specific infectious disease caused by mycobacterium tuberculosis. The disease primarily affects lungs but can affect any of the organ systems, like intestine, meninges, bones and joints, lymph nodes, skin and other tissues of the body.[2]

Tuberculosis remains a worldwide public health problem despite the fact that the causative organism was discovered more than a century ago and highly effective drugs are available for making tuberculosis a preventive and curable disease.

Developed countries have achieved spectacular results in the control of tuberculosis. The problem is more acute in developing countries. Tuberculosis continues to be one of the major public health problems in India; eight out of ten people who contract tuberculosis are in the economically productive age group of 15 to 49 years.[2]

Tuberculosis is a social disease with medical aspects described as barometer of social welfare. The social factors are poverty, illiteracy, ignorance, poor housing, over crowding, population explosion, under nutrition, lack of awareness about illness. All these factors are interrelated and contribute to occurrence of the disease.[2]

## Objectives

1. To study the various clinical presentations of cases diagnosed clinically as abdominal tuberculosis, which compels the resection of the intestine.
2. To illustrate, discuss and describe the various histopathological features of the lesions in these resected intestinal specimens.
3. Clinicopathological correlation of these lesions.

## MATERIALS AND METHODS

All surgically resected specimens diagnosed/suspected clinically as abdominal tuberculosis received from Karnataka Institute of Medical Sciences (KIMS), Hubli hospital and referred to Dept. of Pathology were included and studied in detail. The clinical findings were collected from hospital case sheets or from case records in the hospital record section. The details of gross examination findings were noted. Tissues selected for histopathological examination were processed routinely. Sections of 3 - 6 microns were cut from paraffin blocks and stained with hematoxyllin and eosin. ZN stain was also done to appreciate AFB.

Statistical analysis of various intestinal lesions in relation to age, sex, signs and symptoms, clinical diagnosis and histopathological diagnosis was done.

## RESULTS

We studied 100 cases of resected intestinal specimens during the study period (Sept.2002–Dec. 2003). We totally encountered 22 request forms with clinical suspicion of ileoceocal tuberculosis, out of which only 6 cases proved histologically as cases of TB; the rest were turned out to be ischemic enteritis (nine cases), chronic nonspecific enteritis (presented clinically as acute intestinal obstruction) where no specific lesion is made out (four cases), caecal carcinoma (one case), Crohn’s disease (one case) and intussusception (one case). All cases were of ileoceocal region suggesting the most common site of disease presentation.

### On physical examination

Abdominal tenderness was noted in all the cases along with mass in ileoceocal region, suggesting that these two are the most common signs. None of our cases had pulmonary signs in the past or during admission, and X-ray of chest did not reveal evidence of tuberculosis suggesting a diagnosis of ‘primary intestinal tuberculosis’ in all. In the present study, routine plain X-ray of abdomen was done preoperatively. Out of six patients, two patients presenting with obstructive symptoms (33.33%) had multiple air /fluid levels. Only one patient with perforation had pneumoperitoneum (16.66%).

### Operative procedures

All six cases in the present study were treated medically with antitubercular regimen and surgically with limited ileoceocal resection. One of the two cases presenting with acute intestinal obstruction had a perforation, with co-existent tuberculous peritonitis and associated ascites. Ileoceocal region was involved in all cases (100%).

The single most common finding was of stricture seen in all the cases. Multiple strictures were noted in two cases. Enlarged mesenteric lymph nodes were noted in two cases. Ileoceocal mass occurred in all cases with adherent mesentery. Lysis of adhesions with ileoceocal resection was carried out.

### Grossly

**Figure 1:**
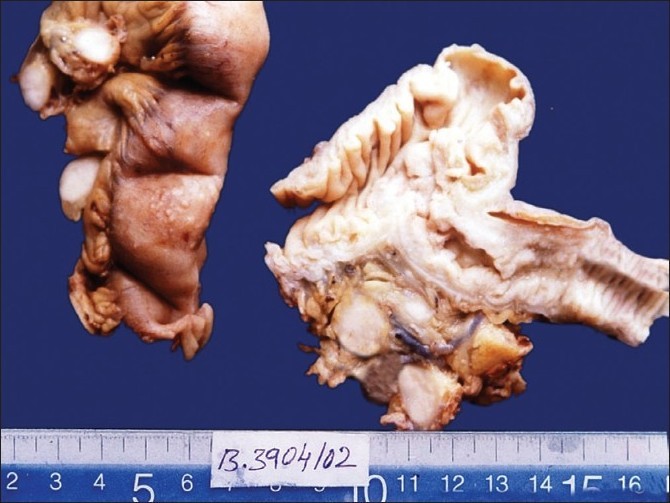
Gross specimen: Resected small intestine showing multiple tubercles scattered on serosal surface with lymph node involvement in cases of ileoceocal tuberculosis

Ileum in all cases showed one or more strictures with dilated adjacent loops. Ileoceocal junction showed stenosis in one case. Caecum and ascending colon showed marked thickening and shortening with scattered tubercles on the serosal surface with enlarged mesenteric lymph nodes.[Figure 1]. When cut open, single to multiple mucosal ulcers were seen in all cases, mostly limited to the terminal ileum. The healed ulcers had resulted in strictures with narrowing of the lumen.

### Microscopy

**Figure 2:**
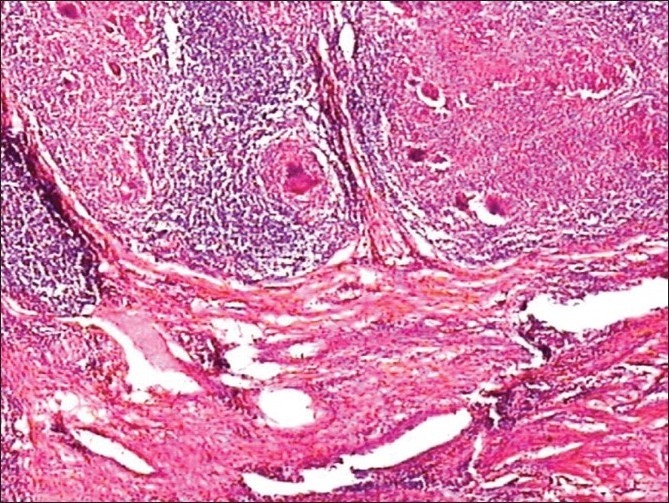
Microscopy showing epitheloid cell granulomas with areas of necrosis (H and E stain, 10 × magnification)

Examination of tissue blocks taken from diseased area of ileum and caecum showed submucosal granulomas containing epitheloid cells, few to many multinucleated Langhan’s cells, in some with central caseous necrosis [Figure 2]. The tubercular granulomas were seen extending to muscular layer in two cases. Serosa also showed similar findings. Perigranuloma lymphocytic infiltration was quite evident. The lymphoid tissue was richest in places where Peyer’s patches probably originally existed. Many sections of mesenteric lymph nodes showed tubercular granulomas with caseation necrosis.

The postoperative course was uneventful in all the cases and patients were discharged with treatment protocol of 9-12 months antitubercular regimens.

## DISCUSSION

The present study differs from previous studies in the aspect that all the resected specimens with clinical suspicion of tuberculosis, irrespective of age and sex, have been studied in detail to investigate the underlying etiology by detailed histopathological examination. Out of a hundred resected specimens, six cases were diagnosed as ileoceocal tuberculosis in the present study. Similar study of 58 cases of primary ulcerohypertrophic ileoceocal tuberculosis was done by Paustian *et al*. The classical ulcerative, hypertrophic and ulcerohypertrophic variants, which were based on the gross morphology of the disease process, were also seen in the present study.[3]

Paustian studied 58 cases of primary ulcerohypertrophic ileoceocal tuberculosis, where none had pulmonary tuberculosis during the period of observation or in the past. None of our cases had pulmonary signs in the past or during admission, and X-ray of chest did not reveal evidence of tuberculosis suggesting a diagnosis of ‘primary intestinal tuberculosis’ in all.[3] They also had no family history of tuberculosis.

Operative diagnoses of tubercular enteritis in many instances were finally diagnosed as ischemic enteritis in nine cases; presence of strictures in ischemic enteritis caused us to erroneously diagnose these cases initially as tuberculosis. We also observed that the presence of mucosal fibrinopurulent exudates that are commonly seen in ischemic enteritis should not be confused with the tubercles of tuberculosis. Sometimes, a small tumor involving ileoceocal valve can cause narrowing of the lumen and patient may present with obstructive signs and symptoms. Another case was diagnosed clinically and grossly as fibrous stricture of tubercular origin and microscopically shown as adenocarcinoma of the caecum. Similarly, a case diagnosed as tuberculosis due to the presence of multiple strictures was diagnosed histologically as Crohn’s disease, demonstrating that other disease entities can also mimic tuberculosis, clinically as well as morphologically.[3] The presence of caseating granulomas propria strongly suggests intestinal tuberculosis, whereas non-caseating mucosal granulomas are commonly seen in Crohn’s disease.One case was presented with mass in iliac region; pre-operative diagnosis of ileoceocal mass of tubercular origin was made. Grossly, intussusception was diagnosed, with a polypoidal mass as a leading point. Microscopically, the polyp was a benign smooth muscle gastrointestinal stromal tumor (GIST) i.e., leiomyoma. Four of our cases presented with acute intestinal obstruction, where pre and intra-operative diagnosis of intestinal tuberculosis due to the presence of strictures were made. On gross examination, multiple mucosal ulcers and strictures were noted. Multiple sections from stricture area and ulcer area failed to demonstrate tubercular granulomas; and the diagnoses of chronic nonspecific enteritis were made.

The present study draws attention to the occasional tragic outcome in patients with this curable disease. Marshal *et al* quotes that, even in centers of excellence, early diagnosis and appropriate treatment of intestinal tuberculosis is frequently delayed because of nonspecific and deceptive clinical presentation of the disease.[4]

Ileoceocal resection has several advantages over the standard hemicolectomy as quoted by Bhansali:[5]

- The extent of dissection and resection is less and consequently the blood loss is minimal.
- Only a small raw area is left retroperitoneally which can easily be retroperitonealised; it takes less time.
- Presence of functioning ascending colon, hepatic flexure and right 1/3 ^rd^ to ½ of transverse colon; the incidence of post-operative diarrhoea is considerably diminished.[5]

## CONCLUSIONS

- An increased awareness of intestinal tuberculosis coupled with varied clinical presentations, nonspecific signs and symptoms, knowledge of pathophysiology, difficulties in diagnostic methods and need of early and specific treatment should improve the outcome for patients with this disease.[1]
- Tuberculosis can no longer be considered as a rare disease, due in part to the AIDS epidemic.
- High index of suspicion must be maintained to ensure timely diagnosis and treatment.[6] The abdominal CT is useful for the diagnosis of abdominal tuberculosis; it reveals asymmetric bowel thickening and enlarged nodes with low attenuation which are the common findings in tuberculous ileitis.
- Intestinal tuberculosis needs to be considered in the differential diagnosis when patients with intestinal pathology are encountered.[7]
